# Supplementary material for: An end-to-end attention-based approach for learning on graphs
Source: Nat Commun. 2025 Jun 5;16:5244. doi: 10.1038/s41467-025-60252-z (PMC12141427; doi:10.1038/s41467-025-60252-z)
Supplement: Supplementary file 1 — Supplementary Information [file 41467_2025_60252_MOESM1_ESM.pdf]

An end-to-end attention-based approach for learning on graphs
Supplementary Information

David Buterez[1,*], Jon Paul Janet[2], Dino Oglic[3], and Pietro Liò[1]

[1]Department of Computer Science and Technology, University of Cambridge, Cambridge, UK
[2]Molecular AI, BioPharmaceuticals R&D, AstraZeneca, Gothenburg, Sweden
[3]Centre for AI, BioPharmaceuticals R&D, AstraZeneca, Cambridge, UK
[*]Corresponding author (db804@cantab.ac.uk)

SI 1 ESA compared to GAT and SAN

GAT

Our masked self-attention module relies on masking within classical scaled dot product attention which comes with different projection matrices for keys, queries, and values. Additionally, we wrap the masked scaled dot product attention with layer normalisation and skip connections, the output of which is passed through an MLP. The latter is not done classically in GAT as part of its attention modules nor in the overall architecture. In contrast to GAT that operates over nodes, our masking operator is defined over sets of edges that are connected if they share a node in common. When it comes to the readout, GAT uses sum, mean or max and aggregates over nodes whereas our architecture relies on pooling by multi-head attention and aggregation over learnt representations of seed vectors inherent to that module. In terms of the encoder that is responsible for learning representations of set items, ours interleaves masked and self-attention layers vertically, whereas in GATs the stacked layers are entirely based on neighbourhood attention.

SAN

One of the main differences in relation to SAN's architecture is the horizontal versus vertical combination of masked and self-attention. Although it is possible to set $\gamma = 0$ for some layers and $\gamma = 1$ for others, this avenue has never been explored in SAN and it would still result in a slightly different encoder as the query and key projections are different for the input graph and its complement. The empirical analysis in [1] also does not provide insights on vertical combinations of masked and self-attention, nor on the impact of attention pooling which is a differentiating factor and an important part of our architecture.

SI 2 Node masking algorithm

The following algorithm illustrates the masking operation on nodes instead of edges.

Algorithm 2: Node masking in the PyTorch Geometric framework.

```
from torch_geometric import unbatch_edge_index
function node_mask(batched_edge_index, batch_map, B, M)
    # batched_edge_index batches all edge_index tensors into a single tensor
    # batch_map maps nodes to graphs
    # B, M are the batch, respectively mask size
    mask ← torch.full(size=(B, M, M), fill=False)
    graph_idx ← batch_map.index_select(0, batched_edge_index[0, :])
    edge_index_list ← unbatch_edge_index(batched_edge_index, batch_map)
    edge_index ← torch.cat(edge_index_list, dim=1)
    mask[graph_idx, edge_index[0, :], edge_index[1, :]] ← True
    return ~mask
```

SI 3 Additional benchmarking results

Supplementary Table 1: The root mean squared error on QM9 (RMSE is the standard for quantum mechanics) and the R^2 for DOCKSTRING (DOCK) and MOLECULENET (MN), presented as mean ± standard deviation over 5 runs, and including GCN and GIN. The mean absolute error (MAE) is reported for PCQM4MV2 over a single run due to the size of the dataset and the field standards. The lowest MAEs and RMSEs and highest R^2 values are highlighted in bold. OOM denotes out-of-memory errors.

	Target	GCN	GIN	DropGIN	GAT	GATv2	PNA	Graphormer	TokenGT	GPS	ESA
QM9 (↓)	μ	0.629 ± 0.005	0.550 ± 0.011	0.552 ± 0.010	0.552 ± 0.011	0.545 ± 0.008	**0.535 ± 0.008**	0.627 ± 0.014	0.755 ± 0.025	0.945 ± 0.174	0.564 ± 0.004
	α	0.525 ± 0.051	0.438 ± 0.020	0.445 ± 0.025	0.479 ± 0.020	0.460 ± 0.018	0.476 ± 0.070	0.404 ± 0.017	0.447 ± 0.014	0.605 ± 0.130	**0.398 ± 0.003**
	ϵ_{HOMO}	0.128 ± 0.005	0.104 ± 0.001	0.106 ± 0.002	0.107 ± 0.002	0.104 ± 0.001	**0.099 ± 0.001**	0.107 ± 0.002	0.123 ± 0.003	0.113 ± 0.003	0.103 ± 0.003
	ϵ_{LUMO}	0.136 ± 0.002	0.120 ± 0.004	0.123 ± 0.004	0.124 ± 0.003	0.127 ± 0.001	**0.109 ± 0.001**	0.112 ± 0.001	0.130 ± 0.003	0.108 ± 0.002	0.114 ± 0.001
	$\Delta\epsilon$	0.184 ± 0.006	0.164 ± 0.004	0.166 ± 0.004	0.163 ± 0.006	0.163 ± 0.003	**0.139 ± 0.001**	0.163 ± 0.004	0.177 ± 0.006	0.154 ± 0.006	0.152 ± 0.001
	$\langle R^2 \rangle$	33.913 ± 0.922	29.925 ± 0.302	29.870 ± 0.343	30.911 ± 0.152	30.149 ± 0.360	28.503 ± 0.444	29.628 ± 0.351	31.540 ± 0.422	30.421 ± 1.192	**28.328 ± 0.321**
	ZPVE	0.035 ± 0.001	0.032 ± 0.001	0.034 ± 0.002	0.033 ± 0.001	0.032 ± 0.001	0.032 ± 0.003	0.059 ± 0.031	0.030 ± 0.001	0.033 ± 0.006	**0.026 ± 0.001**
	U_0	30.971 ± 6.472	42.024 ±17.272	29.817 ± 6.322	27.823 ± 3.981	25.405 ± 3.617	31.644 ± 6.493	24.600 ± 4.699	15.477 ± 4.675	14.496 ± 4.342	**4.777 ± 0.671**
	U	25.276 ± 4.471	25.098 ± 5.972	24.741 ± 6.311	30.914 ± 2.620	24.919 ± 4.183	25.038 ± 5.479	15.546 ± 8.228	12.449 ± 1.864	15.820 ± 4.262	**6.799 ± 2.809**
	H	30.924 ± 5.369	24.746 ± 2.314	34.019 ± 5.663	28.924 ± 4.108	23.422 ± 2.767	27.338 ± 8.167	19.006 ± 4.645	11.418 ± 3.716	12.923 ± 4.287	**6.018 ± 1.324**
	G	26.138 ±10.078	26.942 ± 2.910	25.412 ± 4.863	31.494 ± 1.857	23.240 ± 2.535	22.308 ± 3.453	31.198 ± 4.614	29.721 ± 8.790	13.081 ± 4.806	**6.104 ± 1.334**
	c_{V}	0.221 ± 0.014	0.187 ± 0.008	0.195 ± 0.012	0.191 ± 0.005	0.190 ± 0.002	0.179 ± 0.009	0.177 ± 0.023	0.180 ± 0.004	0.190 ± 0.027	**0.158 ± 0.001**
	U_0^{ATOM}	0.371 ± 0.017	0.391 ± 0.035	0.378 ± 0.022	0.392 ± 0.035	0.384 ± 0.021	0.353 ± 0.018	0.289 ± 0.004	0.304 ± 0.021	0.330 ± 0.051	**0.241 ± 0.006**
	U^{ATOM}	0.390 ± 0.029	0.377 ± 0.029	0.404 ± 0.044	0.483 ± 0.072	0.397 ± 0.036	0.361 ± 0.020	0.302 ± 0.014	0.302 ± 0.004	0.334 ± 0.058	**0.243 ± 0.005**
	H^{ATOM}	0.396 ± 0.025	0.370 ± 0.026	0.376 ± 0.038	0.383 ± 0.021	0.406 ± 0.062	0.373 ± 0.029	0.301 ± 0.012	0.312 ± 0.014	0.363 ± 0.089	**0.245 ± 0.004**
	G^{ATOM}	0.368 ± 0.008	0.385 ± 0.021	0.372 ± 0.036	0.342 ± 0.018	0.329 ± 0.009	0.314 ± 0.021	0.261 ± 0.005	0.273 ± 0.006	0.360 ± 0.075	**0.225 ± 0.015**
	A	0.979 ± 0.049	1.317 ± 0.386	0.904 ± 0.059	0.972 ± 0.122	1.078 ± 0.161	1.007 ± 0.101	64.877 ± 29.771	3.823 ± 2.052	1.422 ± 0.437	**0.746 ± 0.106**
	B	0.295 ± 0.004	0.196 ± 0.047	0.194 ± 0.050	0.211 ± 0.038	0.264 ± 0.010	0.256 ± 0.024	0.102 ± 0.028	0.109 ± 0.013	0.158 ± 0.052	**0.079 ± 0.011**
	C	0.284 ± 0.001	0.167 ± 0.062	0.269 ± 0.016	0.266 ± 0.006	0.276 ± 0.004	0.277 ± 0.012	0.115 ± 0.037	0.097 ± 0.025	0.124 ± 0.046	**0.050 ± 0.012**
DOCK (↑)	ESR2	0.642 ± 0.003	0.668 ± 0.003	0.675 ± 0.003	0.666 ± 0.002	0.655 ± 0.004	0.696 ± 0.002	OOM	0.641 ± 0.008	0.676 ± 0.002	**0.697 ± 0.001**
	F2	0.878 ± 0.001	0.887 ± 0.002	0.886 ± 0.001	0.886 ± 0.001	0.885 ± 0.002	0.891 ± 0.002	OOM	0.872 ± 0.006	0.879 ± 0.004	**0.891 ± 0.000**
	KIT	0.814 ± 0.002	0.833 ± 0.001	0.835 ± 0.002	0.833 ± 0.000	0.826 ± 0.001	**0.843 ± 0.001**	OOM	0.800 ± 0.009	0.832 ± 0.001	0.841 ± 0.001
	PARP1	0.912 ± 0.001	0.922 ± 0.001	0.920 ± 0.002	0.921 ± 0.001	0.919 ± 0.001	0.924 ± 0.001	OOM	0.907 ± 0.005	0.915 ± 0.005	**0.925 ± 0.000**
	PGR	0.658 ± 0.004	0.696 ± 0.001	0.702 ± 0.002	0.681 ± 0.005	0.666 ± 0.006	0.717 ± 0.003	OOM	0.684 ± 0.010	0.703 ± 0.009	**0.725 ± 0.003**
MN (↑)	FSOLV	0.957 ± 0.008	0.964 ± 0.007	0.972 ± 0.005	0.959 ± 0.009	0.970 ± 0.007	0.951 ± 0.008	0.927 ± 0.005	0.930 ± 0.016	0.861 ± 0.032	**0.977 ± 0.001**
	LIPO	0.800 ± 0.007	0.819 ± 0.006	0.809 ± 0.007	0.820 ± 0.012	0.821 ± 0.008	**0.830 ± 0.006**	0.607 ± 0.043	0.545 ± 0.022	0.790 ± 0.004	0.809 ± 0.007
	ESOL	0.936 ± 0.005	0.938 ± 0.010	0.935 ± 0.011	0.930 ± 0.006	0.928 ± 0.005	0.942 ± 0.006	0.908 ± 0.018	0.892 ± 0.032	0.911 ± 0.003	**0.944 ± 0.002**

Supplementary Table 2: The table reports the mean absolute error (MAE) and average precision (AP) for two long-range molecular benchmarks involving peptides. The molecular graph of a peptide is much larger than that of a small drug-like molecule and this makes the tasks well-suited for long-range benchmarking. All the results except the ones for ESA and TokenGT are extracted from [2]. The number of layers for PEPT-STRUCT, respectively PEPT-FUNC is given as (·/·).

**Dataset**	**GCN** (6/6)	**GIN** (10/8)	**GPS** (8/6)	**TokenGT** (10/10)	**ESA** (3/4)
PEPT-STR (MAE ↓)	0.2460 ± 0.0007	0.2473 ± 0.0017	0.2509 ± 0.0014	0.2489 ± 0.0013	**0.2453 ± 0.0003**
PEPT-FN (AP ↑)	0.6860 ± 0.0050	0.6621 ± 0.0067	0.6534 ± 0.0091	0.6263 ± 0.0117	**0.7071 ± 0.0015**

Supplementary Table 3: The mean absolute error (MAE) on ZINC, presented as mean ± standard deviation over 5 runs. The lowest values are highlighted in bold.

Dataset (↓)	GCN	GIN	GAT	GATv2	PNA	Graphormer	TokenGT	GPS	ESA	ESA (PE)
ZINC	0.152 ± 0.02	0.068 ± 0.00	0.078 ± 0.01	0.079 ± 0.00	0.057 ± 0.01	0.036 ± 0.00	0.047 ± 0.01	0.024 ± 0.01	0.027 ± 0.00	**0.017 ± 0.00**

Supplementary Table 4: Transfer learning performance (RMSE) on QM9 for HOMO and LUMO, presented as mean ± standard deviation over 5 different runs, including GCN and GIN. All models use the 3D atomic coordinates and atom types as inputs and no other node or edge features. 'Strat.' stands for strategy and specifies the type of learning: GW only (no transfer learning), inductive, or transductive. The lowest values are highlighted in bold.

Task	Strat.	GCN	GIN	DropGIN	GAT	GATv2	PNA	Grph.	TokenGT	GPS	ESA
HOMO	GW	0.171 ± 0.004	0.162 ± 0.002	0.162 ± 0.002	0.159 ± 0.003	0.157 ± 0.002	**0.151 ± 0.001**	0.179 ± 0.009	0.200 ± 0.008	0.162 ± 0.002	0.152 ± 0.003
	Ind.	0.143 ± 0.004	0.138 ± 0.001	0.136 ± 0.000	0.131 ± 0.001	0.133 ± 0.003	0.132 ± 0.002	0.134 ± 0.000	0.156 ± 0.000	0.151 ± 0.002	**0.131 ± 0.000**
	Trans.	0.131 ± 0.001	0.125 ± 0.001	0.126 ± 0.002	0.123 ± 0.001	0.124 ± 0.001	0.121 ± 0.001	0.125 ± 0.001	0.137 ± 0.000	0.147 ± 0.002	**0.119 ± 0.000**
LUMO	GW	0.181 ± 0.002	0.180 ± 0.002	0.180 ± 0.002	0.181 ± 0.002	0.178 ± 0.002	0.174 ± 0.004	0.190 ± 0.006	0.204 ± 0.006	0.178 ± 0.002	**0.174 ± 0.001**
	Ind.	0.161 ± 0.001	0.159 ± 0.001	0.159 ± 0.001	0.156 ± 0.001	0.157 ± 0.001	0.156 ± 0.002	0.151 ± 0.001	0.165 ± 0.000	0.167 ± 0.001	**0.150 ± 0.001**
	Trans.	0.159 ± 0.002	0.155 ± 0.001	0.157 ± 0.001	0.153 ± 0.001	0.153 ± 0.001	0.153 ± 0.001	0.147 ± 0.001	0.156 ± 0.000	0.169 ± 0.001	**0.146 ± 0.000**

Supplementary Table 5: Matthews correlation coefficient (MCC) for graph-level molecular classification tasks – MoleculeNet and National Cancer Institute (NCI), presented as mean ± standard deviation over 5 different runs, and including GCN and GIN. OOM denotes out-of-memory errors. The highest mean values are highlighted in bold.

	Data (↑)	GCN	GIN	DropGIN	GAT	GATv2	PNA	Graphormer	TokenGT	GPS	ESA
MN	BBBP	0.674 ± 0.034	0.704 ± 0.028	0.685 ± 0.017	0.744 ± 0.012	0.728 ± 0.032	0.731 ± 0.028	0.552 ± 0.012	0.578 ± 0.065	0.705 ± 0.044	**0.835 ± 0.014**
	BACE	0.631 ± 0.028	0.646 ± 0.013	0.654 ± 0.034	0.632 ± 0.018	0.645 ± 0.026	0.638 ± 0.017	0.522 ± 0.020	0.578 ± 0.033	0.618 ± 0.032	**0.721 ± 0.019**
	HIV	0.448 ± 0.035	0.408 ± 0.060	0.458 ± 0.028	0.421 ± 0.061	0.337 ± 0.059	0.417 ± 0.045	OOM	0.455 ± 0.017	0.247 ± 0.211	**0.533 ± 0.012**
NCI	NCI1	0.682 ± 0.013	0.694 ± 0.017	0.686 ± 0.027	0.701 ± 0.018	0.646 ± 0.029	0.697 ± 0.025	0.540 ± 0.025	0.532 ± 0.034	0.697 ± 0.027	**0.755 ± 0.012**
	NCI109	0.665 ± 0.024	0.684 ± 0.015	0.681 ± 0.021	0.658 ± 0.008	0.664 ± 0.015	0.670 ± 0.018	0.504 ± 0.022	0.453 ± 0.029	0.623 ± 0.014	**0.700 ± 0.010**

Supplementary Table 6: Accuracy for graph-level molecular classification tasks – MoleculeNet and National Cancer Institute (NCI), presented as mean ± standard deviation over 5 different runs, and including GCN and GIN. OOM denotes out-of-memory errors. The highest mean values are highlighted in bold.

	Data (↑)	GCN	GIN	DropGIN	GAT	GATv2	PNA	Graphormer	TokenGT	GPS	ESA
MN	BBBP	0.871 ± 0.012	0.882 ± 0.010	0.875 ± 0.006	0.898 ± 0.004	0.892 ± 0.012	0.893 ± 0.010	0.826 ± 0.005	0.832 ± 0.022	0.883 ± 0.017	**0.932 ± 0.007**
	BACE	0.813 ± 0.014	0.820 ± 0.007	0.824 ± 0.017	0.813 ± 0.009	0.820 ± 0.012	0.817 ± 0.009	0.758 ± 0.011	0.786 ± 0.015	0.804 ± 0.017	**0.858 ± 0.010**
	HIV	0.974 ± 0.001	0.973 ± 0.001	0.974 ± 0.001	0.973 ± 0.002	0.971 ± 0.001	0.973 ± 0.001	OOM	0.973 ± 0.001	0.971 ± 0.003	**0.976 ± 0.001**
NCI	NCI1	0.842 ± 0.006	0.848 ± 0.008	0.843 ± 0.014	0.851 ± 0.010	0.824 ± 0.015	0.850 ± 0.012	0.770 ± 0.012	0.767 ± 0.018	0.850 ± 0.014	**0.878 ± 0.006**
	NCI109	0.831 ± 0.011	0.842 ± 0.007	0.840 ± 0.010	0.826 ± 0.005	0.831 ± 0.007	0.834 ± 0.009	0.749 ± 0.011	0.721 ± 0.017	0.809 ± 0.006	**0.850 ± 0.005**

Supplementary Table 7: Matthews correlation coefficient (MCC) for graph-level classification tasks from various domains, presented as mean ± standard deviation over 5 different runs, and including GCN and GIN. OOM denotes out-of-memory errors, and N/A that the model is unavailable (e.g. node/edge features are not integers, which are required for Graphormer and TokenGT). The highest mean values are highlighted in bold.

	**Dataset** (↑)	**GCN**	**GIN**	**DropGIN**	**GAT**	**GATv2**	**PNA**	**Graphormer**	**TokenGT**	**GPS**	**ESA**
	MALNETTINY	0.896 ± 0.006	0.903 ± 0.005	0.902 ± 0.006	0.899 ± 0.006	0.902 ± 0.007	0.915 ± 0.008	OOM	0.777 ± 0.008	0.795 ± 0.011	**0.931 ± 0.001**
Vis.	MNIST	0.957 ± 0.002	0.965 ± 0.004	0.970 ± 0.001	0.972 ± 0.002	0.979 ± 0.002	0.978 ± 0.003	N/A	N/A	0.980 ± 0.001	**0.986 ± 0.000**
	CIFAR10	0.621 ± 0.003	0.614 ± 0.006	0.614 ± 0.009	0.659 ± 0.009	0.662 ± 0.011	0.686 ± 0.005	N/A	N/A	0.708 ± 0.005	**0.727 ± 0.003**
Bio.	ENZYMES	0.695 ± 0.048	0.632 ± 0.046	0.576 ± 0.039	0.748 ± 0.020	0.744 ± 0.023	0.684 ± 0.034	N/A	N/A	0.734 ± 0.045	**0.751 ± 0.009**
	PROTEINS	0.419 ± 0.037	0.421 ± 0.036	0.459 ± 0.005	0.463 ± 0.036	0.490 ± 0.042	0.467 ± 0.067	N/A	N/A	0.443 ± 0.022	**0.589 ± 0.017**
	DD	0.546 ± 0.058	0.539 ± 0.032	0.537 ± 0.070	0.465 ± 0.049	0.530 ± 0.034	0.559 ± 0.080	OOM	0.459 ± 0.049	0.605 ± 0.041	**0.652 ± 0.030**
Synth	SYNTH	1.000 ± 0.000	1.000 ± 0.000	1.000 ± 0.000	1.000 ± 0.000	1.000 ± 0.000	1.000 ± 0.000	N/A	N/A	1.000 ± 0.000	**1.000 ± 0.000**
	SYNT N.	0.699 ± 0.029	0.910 ± 0.030	0.975 ± 0.051	0.761 ± 0.067	0.909 ± 0.067	1.000 ± 0.000	N/A	N/A	1.000 ± 0.000	**1.000 ± 0.000**
	SYNTHIE	0.935 ± 0.054	0.930 ± 0.045	0.942 ± 0.024	0.701 ± 0.045	0.798 ± 0.038	0.879 ± 0.058	N/A	N/A	**0.951 ± 0.019**	0.947 ± 0.016
Social	IMDB-B	0.603 ± 0.056	0.537 ± 0.199	0.608 ± 0.061	0.688 ± 0.037	0.600 ± 0.049	0.563 ± 0.067	0.565 ± 0.045	0.606 ± 0.052	0.598 ± 0.045	**0.738 ± 0.026**
	IMDB-M	0.216 ± 0.044	0.119 ± 0.079	0.117 ± 0.099	0.203 ± 0.052	0.202 ± 0.035	0.034 ± 0.068	0.222 ± 0.024	0.202 ± 0.023	0.232 ± 0.021	**0.247 ± 0.034**
	TWITCH E.	0.386 ± 0.006	0.358 ± 0.023	0.379 ± 0.011	0.373 ± 0.011	0.371 ± 0.011	0.078 ± 0.159	0.387 ± 0.003	0.393 ± 0.001	0.395 ± 0.001	**0.398 ± 0.000**
	REDDIT THR.	0.556 ± 0.007	0.556 ± 0.011	0.556 ± 0.007	0.533 ± 0.021	0.536 ± 0.023	0.113 ± 0.227	0.567 ± 0.003	0.564 ± 0.001	**0.568 ± 0.003**	0.568 ± 0.002

Supplementary Table 8: Accuracy for graph-level classification tasks from various domains, presented as mean ± standard deviation over 5 different runs, and including GCN and GIN. OOM denotes out-of-memory errors, and N/A that the model is unavailable (e.g. node/edge features are not integers, which are required for Graphormer and TokenGT). The highest mean values are highlighted in bold.

	**Dataset (↑)**	**GCN**	**GIN**	**DropGIN**	**GAT**	**GATv2**	**PNA**	**Graphormer**	**TokenGT**	**GPS**	**ESA**
	MALNETTINY	0.916 ± 0.005	0.922 ± 0.004	0.921 ± 0.005	0.918 ± 0.005	0.921 ± 0.006	0.931 ± 0.006	OOM	0.820 ± 0.007	0.835 ± 0.009	**0.944 ± 0.001**
Vis.	MNIST	0.961 ± 0.001	0.969 ± 0.004	0.973 ± 0.001	0.975 ± 0.002	0.981 ± 0.001	0.980 ± 0.003	N/A	N/A	0.982 ± 0.001	**0.988 ± 0.000**
	CIFAR10	0.659 ± 0.003	0.652 ± 0.005	0.652 ± 0.008	0.693 ± 0.008	0.695 ± 0.010	0.717 ± 0.005	N/A	N/A	0.737 ± 0.005	**0.754 ± 0.002**
Bio.	ENZYMES	0.735 ± 0.039	0.683 ± 0.037	0.651 ± 0.037	0.786 ± 0.014	0.780 ± 0.019	0.730 ± 0.022	N/A	N/A	0.777 ± 0.039	**0.794 ± 0.015**
	PROTEINS	0.755 ± 0.015	0.755 ± 0.017	0.768 ± 0.000	0.768 ± 0.015	0.777 ± 0.020	0.777 ± 0.029	N/A	N/A	0.768 ± 0.011	**0.827 ± 0.007**
	DD	0.782 ± 0.031	0.773 ± 0.020	0.782 ± 0.033	0.731 ± 0.031	0.760 ± 0.020	0.790 ± 0.039	OOM	0.739 ± 0.030	0.808 ± 0.017	**0.835 ± 0.016**
Synth	SYNTH	1.000 ± 0.000	1.000 ± 0.000	1.000 ± 0.000	1.000 ± 0.000	1.000 ± 0.000	1.000 ± 0.000	N/A	N/A	1.000 ± 0.000	**1.000 ± 0.000**
	SYNT N.	0.847 ± 0.016	0.953 ± 0.016	0.987 ± 0.027	0.880 ± 0.034	0.953 ± 0.034	1.000 ± 0.000	N/A	N/A	1.000 ± 0.000	**1.000 ± 0.000**
	SYNTHIE	0.956 ± 0.039	0.955 ± 0.033	0.963 ± 0.018	0.776 ± 0.037	0.855 ± 0.031	0.918 ± 0.040	N/A	N/A	0.958 ± 0.014	**0.963 ± 0.012**
Social	IMDB-B	0.802 ± 0.028	0.766 ± 0.097	0.804 ± 0.030	0.842 ± 0.018	0.800 ± 0.024	0.780 ± 0.034	0.780 ± 0.023	0.802 ± 0.026	0.794 ± 0.024	**0.868 ± 0.013**
	IMDB-M	0.476 ± 0.030	0.411 ± 0.052	0.410 ± 0.064	0.470 ± 0.033	0.469 ± 0.024	0.356 ± 0.046	0.484 ± 0.015	0.470 ± 0.015	0.476 ± 0.013	**0.487 ± 0.011**
	TWITCH E.	0.697 ± 0.003	0.682 ± 0.012	0.694 ± 0.005	0.690 ± 0.005	0.690 ± 0.005	0.506 ± 0.098	0.698 ± 0.001	0.701 ± 0.001	0.702 ± 0.000	**0.703 ± 0.000**
	REDDIT THR.	0.778 ± 0.003	0.776 ± 0.005	0.777 ± 0.003	0.765 ± 0.010	0.767 ± 0.011	0.545 ± 0.118	0.782 ± 0.001	0.780 ± 0.001	**0.783 ± 0.001**	0.782 ± 0.001

Supplementary Table 9: Matthews correlation coefficient (MCC) for 11 node-level classification tasks, presented as mean ± standard deviation over 5 different runs, including GIN and DropGIN. The number of nodes for the shortest path (SP) benchmarks is given in parentheses (based on randomly-generated infected Erdős–Rényi (ER) graphs; SI 11 for details). Additional heterophily results are provided in Supplementary Table 11. The highest mean values are highlighted in bold.

	**Dataset (↑)**	**GCN**	**GIN**	**DropGIN**	**GAT**	**GATv2**	**PNA**	**Graphormer**	**TokenGT**	**GPS**	**ESA**
	PPI	0.979 ± 0.002	0.905 ± 0.066	0.785 ± 0.141	**0.990 ± 0.000**	0.982 ± 0.005	0.990 ± 0.000	N/A	N/A	N/A	0.989 ± 0.001
CITE	CITESEER	0.608 ± 0.008	0.587 ± 0.010	0.324 ± 0.017	0.587 ± 0.030	0.613 ± 0.006	0.511 ± 0.033	OOM	0.384 ± 0.022	0.538 ± 0.012	**0.632 ± 0.005**
CITE	CORA	0.767 ± 0.008	0.727 ± 0.010	0.490 ± 0.033	0.748 ± 0.014	0.727 ± 0.010	0.637 ± 0.029	OOM	0.366 ± 0.185	0.643 ± 0.039	**0.768 ± 0.005**
HETEROPHILY	ROMAN EMP.	0.470 ± 0.004	0.791 ± 0.003	0.796 ± 0.002	0.741 ± 0.010	0.763 ± 0.004	0.855 ± 0.002	N/A	N/A	0.837 ± 0.014	**0.869 ± 0.002**
HETEROPHILY	AMAZON R.	0.179 ± 0.003	0.262 ± 0.015	0.228 ± 0.018	0.256 ± 0.009	0.253 ± 0.013	0.206 ± 0.018	N/A	N/A	0.114 ± 0.139	**0.336 ± 0.006**
HETEROPHILY	MINESWEEPER	0.303 ± 0.002	0.563 ± 0.028	0.455 ± 0.162	0.477 ± 0.018	0.512 ± 0.010	0.624 ± 0.043	N/A	N/A	0.564 ± 0.011	**0.688 ± 0.001**
HETEROPHILY	TOLOKERS	0.299 ± 0.011	0.299 ± 0.065	0.340 ± 0.012	0.384 ± 0.009	0.386 ± 0.005	0.350 ± 0.048	N/A	N/A	0.351 ± 0.021	**0.427 ± 0.004**
HETEROPHILY	SQUIRREL	0.197 ± 0.011	0.194 ± 0.026	0.230 ± 0.019	0.237 ± 0.017	0.238 ± 0.013	0.224 ± 0.011	0.104 ± 0.085	0.175 ± 0.017	0.228 ± 0.021	**0.289 ± 0.006**
HETEROPHILY	CHAMELEON	0.324 ± 0.006	0.272 ± 0.024	0.287 ± 0.026	0.240 ± 0.056	0.281 ± 0.030	0.267 ± 0.030	0.253 ± 0.038	0.260 ± 0.045	0.298 ± 0.081	**0.387 ± 0.018**
INF	ER (15K)	0.216 ± 0.016	0.387 ± 0.067	0.244 ± 0.049	0.315 ± 0.001	0.316 ± 0.000	0.543 ± 0.086	OOM	0.065 ± 0.000	0.178 ± 0.040	**0.915 ± 0.007**
INF	ER (30K)	0.094 ± 0.035	0.330 ± 0.021	0.292 ± 0.022	0.102 ± 0.064	0.102 ± 0.058	0.423 ± 0.049	OOM	OOM	OOM	**0.872 ± 0.008**

Supplementary Table 10: Accuracy for 11 node-level classification tasks, presented as mean ± standard deviation over 5 different runs, including GIN and DropGIN. The number of nodes for the shortest path (SP) benchmarks is given in parentheses (based on randomly-generated infected Erdős–Rényi (ER) graphs; SI 11 for details). Additional heterophily results are provided in Supplementary Table 11. The highest mean values are highlighted in bold.

	**Dataset (↑)**	**GCN**	**GIN**	**DropGIN**	**GAT**	**GATv2**	**PNA**	**Graphormer**	**TokenGT**	**GPS**	**ESA**
	PPI	0.991 ± 0.001	0.960 ± 0.027	0.913 ± 0.056	**0.996 ± 0.000**	0.992 ± 0.002	0.996 ± 6.480	N/A	N/A	N/A	0.995 ± 0.000
CITE	CITESEER	0.648 ± 0.008	0.626 ± 0.008	0.426 ± 0.016	0.625 ± 0.018	0.649 ± 0.005	0.557 ± 0.027	OOM	0.470 ± 0.016	0.614 ± 0.011	**0.651 ± 0.008**
CITE	CORA	**0.822 ± 0.005**	0.786 ± 0.012	0.595 ± 0.029	0.803 ± 0.014	0.785 ± 0.007	0.716 ± 0.019	OOM	0.456 ± 0.159	0.698 ± 0.033	0.820 ± 0.004
HETEROPHILY	ROMAN EMP.	0.462 ± 0.002	0.755 ± 0.005	0.764 ± 0.003	0.703 ± 0.016	0.721 ± 0.009	0.831 ± 0.004	N/A	N/A	**0.851 ± 0.013**	0.850 ± 0.007
HETEROPHILY	AMAZON R.	0.284 ± 0.003	0.363 ± 0.013	0.324 ± 0.013	0.358 ± 0.013	0.352 ± 0.017	0.311 ± 0.016	N/A	N/A	0.418 ± 0.061	**0.445 ± 0.016**
HETEROPHILY	MINESWEEPER	0.605 ± 0.001	0.764 ± 0.022	0.702 ± 0.092	0.713 ± 0.016	0.729 ± 0.007	0.801 ± 0.033	N/A	N/A	**0.862 ± 0.002**	0.852 ± 0.003
HETEROPHILY	TOLOKERS	0.595 ± 0.006	0.601 ± 0.034	0.628 ± 0.011	0.649 ± 0.007	0.642 ± 0.005	0.649 ± 0.022	N/A	N/A	**0.805 ± 0.003**	0.714 ± 0.011
HETEROPHILY	SQUIRREL	0.318 ± 0.010	0.318 ± 0.025	0.346 ± 0.024	0.351 ± 0.010	0.353 ± 0.013	0.338 ± 0.018	0.258 ± 0.048	0.294 ± 0.017	**0.422 ± 0.013**	0.409 ± 0.008
HETEROPHILY	CHAMELEON	0.436 ± 0.004	0.399 ± 0.020	0.414 ± 0.019	0.363 ± 0.042	0.400 ± 0.023	0.395 ± 0.021	0.378 ± 0.032	0.381 ± 0.034	0.431 ± 0.068	**0.478 ± 0.015**
INF	ER (15K)	0.227 ± 0.020	0.351 ± 0.116	0.154 ± 0.086	0.361 ± 0.003	0.364 ± 0.000	0.528 ± 0.073	OOM	0.091 ± 0.000	0.626 ± 0.003	**0.886 ± 0.011**
INF	ER (30K)	0.159 ± 0.020	0.189 ± 0.103	0.124 ± 0.078	0.246 ± 0.130	0.237 ± 0.121	0.442 ± 0.087	OOM	OOM	OOM	**0.760 ± 0.040**

Supplementary Table 11: Matthews correlation coefficient (MCC) for the heterophilous node-level classification tasks and two additional baselines, presented as mean ± standard deviation over 5 different runs.

	Dataset (↑)	GraphSAGE	GT	Polynormer
HETEROPHILY	ROMAN EMPIRE	0.83 ± 0.00	0.84 ± 0.00	0.92 ± 0.00
	AMAZON RATINGS	0.36 ± 0.00	0.34 ± 0.01	0.38 ± 0.00
	MINESWEEPER	0.65 ± 0.01	0.57 ± 0.04	0.77 ± 0.02
	TOLOKERS	0.23 ± 0.02	0.38 ± 0.01	0.41 ± 0.02
	SQUIRREL FILTERED	0.14 ± 0.03	0.18 ± 0.01	0.23 ± 0.02
	CHAMELEON FILTERED	0.27 ± 0.03	0.25 ± 0.04	0.39 ± 0.02

SI 4 Additional Exphormer and Polynormer results

We evaluate Exphormer [3], which extends GraphGPS by adding virtual global nodes and expander graphs, and Polynormer [4], which is an interesting combination of graph attention and gating. We perform this additional evaluation on a smaller but representative selection of datasets: *i*) relatively large-scale datasets from computational chemistry and computer vision (ZINC and MNIST), *ii*) small-scale molecular datasets (from MOLECULENET), and *iii*) bioinformatics benchmarks (ENZYMES, PROTEINS).

Polynormer was originally evaluated only on node classification tasks, whereas our main focus is on graph-level predictive performance. We have adapted this baseline to graph-level tasks by including a simple mean pooling layer, and then trained and evaluated tuned Polynormer models.

The results of our evaluation on these tasks, featuring both Polynormer and Exphormer, are provided in Tables 1 and 3. In line with expectations, Exphormer performs similarly to GraphGPS, being better on datasets such as MNIST and PROTEINS, but slightly underperforming on the small molecular datasets and ZINC. Polynormer does not appear to be competitive on several of the larger graph-level datasets, but is otherwise close.

Supplementary Table 12: R^2 for MOLECULENET datasets, presented as mean ± standard deviation over 5 runs. ESA and GPS results are reproduced from Table 1.

	Dataset (↑)	GPS	Exphormer	Polynormer	ESA
MN	FREESOLV	0.86 ± 0.03	0.89 ± 0.01	0.89 ± 0.01	**0.98 ± 0.00**
	LIPO	0.79 ± 0.00	0.75 ± 0.02	0.80 ± 0.01	**0.81 ± 0.01**
	ESOL	0.91 ± 0.00	0.91 ± 0.01	0.90 ± 0.01	**0.94 ± 0.00**

Supplementary Table 13: Matthews correlation coefficient (MCC) for classification datasets, presented as mean ± standard deviation over 5 runs. ESA and GPS results are reproduced from Table 3.

	Dataset (↑)	GPS	Exphormer	Polynormer	ESA
MN	BBBP	0.705 ± 0.04	0.675 ± 0.03	0.736 ± 0.03	**0.835 ± 0.01**
	BACE	0.618 ± 0.03	0.601 ± 0.02	0.612 ± 0.04	**0.721 ± 0.02**
BIO	ENZYMES	0.734 ± 0.05	0.714 ± 0.02	0.628 ± 0.00	**0.751 ± 0.01**
	PROTEINS	0.443 ± 0.02	0.520 ± 0.03	0.572 ± 0.06	**0.589 ± 0.02**
CV	MNIST	0.980 ± 0.00	0.983 ± 0.00	0.970 ± 0.00	**0.986 ± 0.00**

Supplementary Table 14: Mean absolute error (MAE) on the ZINC dataset, presented as mean ± standard deviation over 5 runs. ESA and GPS results are reproduced from Table 1.

Dataset (↓)	GPS	Exphormer	Polynormer	ESA	ESA (PE)
ZINC	0.024 ± 0.01	0.041 ± 0.01	0.101 ± 0.00	0.027 ± 0.00	**0.017 ± 0.00**

SI 5 Additional time and memory results

Continuation of Figure 3, including time and memory results for the DOCKSTRING dataset.

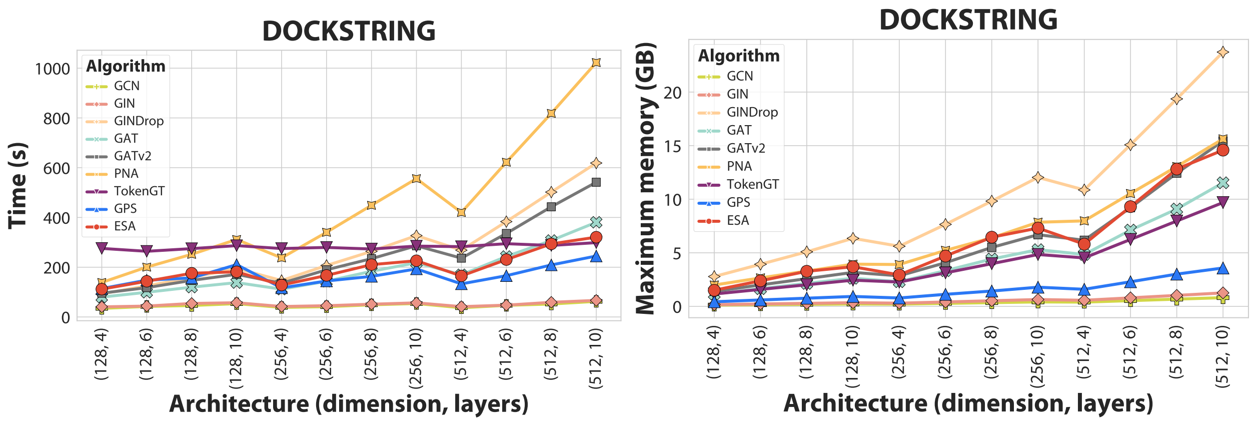

(a) Training time for DOCKSTRING. **(b)** Allocated memory for DOCKSTRING.

Supplementary Figure 1: (a). The elapsed time for training all evaluated models for a single epoch on the DOCKSTRING dataset (in seconds). Graphormer is not included as it runs out of memory. **(b).** The maximum allocated memory during the same training epoch (GB).

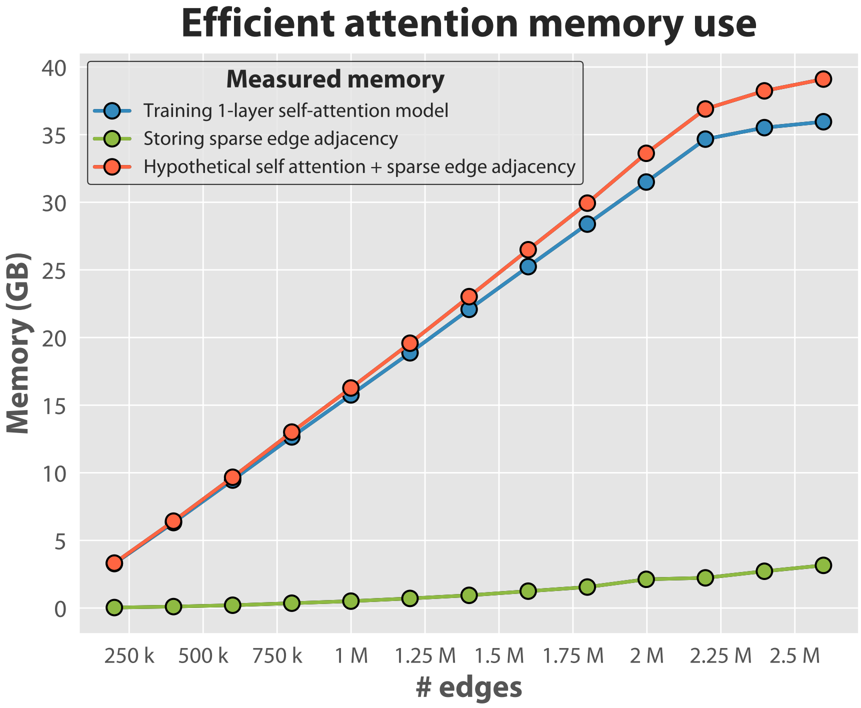

Supplementary Figure 2: Memory usage for efficient attention versus the number of edges.

SI 6 Memory scaling with sparse edge adjacency storage

It is worth noting the efficiency of optimised attention implementations. For a sequence length of 1 million (10^6), an efficient implementation calculates 1 trillion (10^{12}) attention scores without materialising the square attention matrix. To put this in the context of graph learning and ESA, we generated random Barabasi-Albert (BA) graphs using PyTorch Geometric, with varying numbers of edges that range from 200,000 to 2.6 million. We next train a model using a single self-attention layer (no masking) and representative model parameters (total model dimension 256, 8 attention heads, with MLPs and normalisation layers). Supplementary Figure 2 (blue line) illustrates the peak memory use graphically, demonstrating the linear memory scaling (for the largest graphs, the kernel might be adjusting as the GPU memory limit of 40GB approaches).

We then measured and plotted the amount of memory required to store the **sparse edge masking information**, as opposed to a full dense matrix (green line in Supplementary Figure 2). Clearly, this cost is relatively trivial even for graphs as large as 2.6 million edges.

We next hypothesised what an efficient implementation might look like by simply adding the sparse storage cost to the self-attention cost (orange line in Supplementary Figure 2). Note that we are still assuming that full self-attention is being used; however, an efficient implementation could avoid hundreds of millions of unnecessary calculations.

SI 7 Limitations

In terms of limitations, we highlight that the available libraries are not optimised for masking or custom attention patterns. This is most evident for very dense graphs (tens of thousands of edges or more). Memory efficient and Flash attention are natively available in PyTorch [5] starting from version 2.0, as well as in the xFormers library [6]. More specifically, we have tested at least 5 different implementations of ESA: (1) leveraging the `MultiheadAttention` module from PyTorch, (2) leveraging the `MultiHeadDispatch` module from xFormers, (3) a manual implementation of multihead attention, relying on PyTorch's `scaled_dot_product_attention` function, (4) a manual implementation of multihead attention, relying on xFormers' `memory_efficient_attention`, and (5) a naive implementation. Options (1) - (4) can all make use of efficient and fast implementations. However, we have observed performance differences between the 4 implementations as well as compared to a naive implementation. This behaviour is likely due to the different low-level kernel implementations. Moreover, Flash attention does not currently support custom attention masks, as there is little interest for such functionality from a language modelling perspective.

Although masks can be computed efficiently during training, all frameworks require the last two dimensions of the input mask tensor to be of shape (L_n, L_n) for nodes or (L_e, L_e) for edges, effectively squaring the number of nodes or edges. However, the mask tensors are sparse and a sparse tensor alternative could greatly reduce the memory consumption for large and dense graphs. Such an option exists for native PyTorch attention, but it is currently broken. Additionally, even a single boolean requires an entire byte of storage in PyTorch, increasing the memory usage by 8 times compared to the theoretical requirement of 1 bit.

Another possible optimisation would be to use nested (ragged) tensors to represent graphs, since padding is currently necessary to ensure identical dimensions for attention. A prototype nested tensor attention is available in PyTorch; however, not all the required operations are supported, and converting between normal and nested tensors is slow.

For all implementations, it is required that the mask tensor is repeated by the number of attention heads (e.g. 8 or 16). However, a notable bottleneck is encountered for the `MultiheadAttention` and `MultiHeadDispatch` variants described above, which require that the repeats occur in the batch dimension, i.e. requiring 3D mask tensors of shape $(B \times H, L, L)$, where H is the number of heads. The other two efficient implementations require a 4D mask instead, i.e. (B, H, L, L), where one can use PyTorch's `expand` function instead of `repeat`. The `expand` alternative does not use any additional memory, while `repeat` requires $\times H$ memory. Note that it is not possible to reshape the 4D tensor created using `expand` without using additional memory.

Finally, we noticed a limitation involving PyTorch's `nonzero()` tensor method, which is required as part of the edge masking algorithm (Algorithm 1). This is covered in detail in SI 12. Currently, the `nonzero()` method fails for very dense graphs. A fix would require an update to 64-bit integer limits.

SI 8 Helper functions

`consecutive` is a helper function that generates consecutive numbers starting from 0, with a length specified in its tensor argument as the difference between adjacent elements, and a second integer argument used for the last length computation, e.g. `consecutive`$([1, 4, 6], 10) = [0, 1, 2, 0, 1, 0, 1, 2, 3]$, and `first_unique_index` finds the first occurrence of each unique element in the tensor (sorted), e.g. `first_unique_index`$([3, 2, 3, 4, 2]) = [1, 0, 3]$. The implementations are available in our code base.

SI 9 Experimental setup

We follow a simple and universal experimental protocol to ensure that it is possible to compare the results of different methods and to evaluate a large number of datasets with high throughput. In SI 9.1 we describe the grid search approach and the search parameters used. For basic hyperparameters such as batch size and learning rate, we chose a number of reasonable hyperparameters and settings for all methods, regardless of their nature (GNN or attention-based). This includes the AdamW optimiser [7], more specifically the 8-bit version [8], learning rate (0.0001), batch size (128), mixed precision training with the `bfloat16` tensor format, early stopping with a patience of 30 epochs (100 for very small datasets such as FREESOLV), and gradient clipping (set to the default value of 0.5). Furthermore, we used a simple learning rate scheduler that halved the learning rate if no improvement was encountered for 15 epochs (half the early stopping patience). If these parameters led to out-of-memory errors, we attempted reducing the batch size by 2 until the error was fixed, or reducing the hidden dimension as a last resort.

For Graphormer and TokenGT, we leverage the `huggingface` [9] implementation. We have adapted the TokenGT implementation to use Flash attention, which was not originally supported. For Graphormer, this optimisation is not possible since Flash attention is not compatible with some operations required by

Graphormer. However, we did optimise the data loading process compared to the original `huggingface` implementation, leading to lower RAM usage.

SI 9.1 Hyperparameter tuning

For a fair and comprehensive evaluation, we tune each algorithm for each dataset using a grid search approach and a selection of reasonable hyperparameters. These include the number of layers, the number of attention heads for GAT(v2) and graph transformers, dropout, and hidden dimensions. For graph-level GNNs, we evaluated configurations with 4 to 6 layers, hidden dimensions in $\{128, 256, 512\}$, the number of GAT heads in $\{8, 16\}$, and GAT dropout in $\{0, 0.2\}$. For node-level GNNs, we adapted the search since these tasks are more sensitive to overfitting, and used a number of layers in $\{1, 2, 4, 6\}$, hidden dimensions in $\{64, 128, 256\}$, the same GAT heads and dropout settings, and dropout after each GNN layer in $\{0, 0.2\}$. For Graphormer, TokenGT, and GraphGPS, we evaluated models with the number of layers in $\{4, 6, 8, 10\}$, the number of attention heads in $\{4, 8, 16\}$, and hidden dimensions in $\{128, 256, 512\}$. For ESA, we generally focused on models with 6 to 10 layers, with SABs at the start and end and MABs in the middle. The hidden dimensions are selected from $\{256, 512\}$ and the number of attention heads from $\{8, 16, 32\}$. We evaluated pre-LN and post-LN architectures, standard and gated MLPs, and different MLP hidden dimensions and number of MLP layers on a dataset-by-dataset basis. The best configuration is selected based on the validation loss, and results are reported on the test set from 5 different runs. Based on recent reports on the performance of GNNs [10], we augmented all 6 GNN baselines with residual connections and normalisation layers for each graph convolutional layer. These strategies are not part of the original message passing specification, but lead to substantial uplifts. For datasets with established splits, such as CIFAR10, MNIST, or ZINC, we use the available splits. For DOCKSTRING, only train and test splits are available, so we randomly extract 20,000 train molecules for validation. Otherwise, we generate our own splits with a train/validation/test ratio of 80%/10%/10%. All models are trained and evaluated using mixed-precision training with the `bfloat16` tensor format.

SI 9.2 Metrics

Given the scale of our evaluation, it is crucial to use an appropriate selection of performance metrics. To this end, we selected the metrics according to established and recent literature. For classification tasks, there is a growing consensus that Matthew's correlation coefficient (MCC) is the preferred metric over alternatives such as accuracy, F-score, and the area under the receiver operating characteristic curve (AUROC or ROC-AUC) [11–14]. The AUROC in particular has been shown to be problematic [15–17]. The MCC is an informative measure of a classifier's performance as it summarises the four basic rates of a confusion matrix: sensitivity, specificity, precision, and negative predictive value [15]. Similarly, the R^2 has been proven to be more informative than alternatives such as the mean absolute or squared errors for regression tasks [18]. Thus, our first choices for reporting results are the MCC and R^2, depending on the task. For comparison with leaderboard results and for specialised fields such as quantum mechanics, we also report comparable metrics (i.e. accuracy, mean absolute error, or root mean squared error).

SI 9.3 Baselines

We include classic message passing baselines in the form of GCN, GAT, and GIN due to their recent resurgence against sophisticated graph transformers and their widespread use. We also include the improved GATv2 [19] to complement GAT, and PNA for being neglected in other works despite its remarkable empirical performance. We complete the message passing baselines by including DropGNN [20], a family of provably expressive GNNs that can solve tasks beyond 1-WL in an efficient manner by randomly dropping nodes. As in the original paper, we use GIN as the main underlying mechanism and label this technique DropGIN. Regarding transformer baselines, we select Graphormer, TokenGT, and GraphGPS, not only because of their widespread use but also because of generally outperforming previous generation graph transformers such as SAN. This selection is balanced in the sense that Graphormer and TokenGT are part of a class of algorithms that focuses on representing the graph structure through encodings and token identifiers, while GraphGPS relies on GNNs and is thus a hybrid approach.

SI 10 Transfer learning setup

The transfer learning setup consists of randomly selected training, validation, and test sets of 25K, 5K, and, respectively, 10K molecules with GW calculations (from the total of 133,885). This setup mimics the low amounts of high quality/fidelity data available in drug discovery and quantum simulation projects. In the transductive case, the entire dataset with DFT targets is used for pre-training (including the 10K test set compounds, but only with DFT-level measurements), while in the inductive setting the 10K set is completely excluded. Here, we perform transfer learning by pre-training a model on the DFT target for a fixed number of epochs (150) and then fine-tuning it on the subset of 25K GW calculations. In the transductive case,

pre-training occurs on the full set of 133K DFT calculations, while in the inductive case the DFT test set values are removed (note that the evaluation is done on the test set GW measurements).

SI 11 Infected graph generation

The infected graphs are generated using the `InfectionDataset` from PyTorch Geometric. We generate two Erdős–Rényi (ER) graphs with different sizes:

- with 15,000 nodes, 40 infected nodes, a maximum shortest path length of 20, and an edge probability of 0.00009.
- with 30,000 nodes, 20 infected nodes, a maximum shortest path length of 20, and an edge probability of 0.00005.

These settings ensure a relatively balanced classification task for both graph sizes.

SI 12 Adaptations for Open Catalyst Project

Extending a given model to work with 3D data is not trivial, as demonstrated by the follow-up paper dedicated to extending and benchmarking Graphormer on 3D molecular problems [21]. As described in that paper, for the Open Catalyst Project (OCP) data, certain pre-processing steps are taken to ensure satisfactory performance. Concretely, a set of Gaussian basis functions is used to encode atomic distances, which are not used in their raw form. The idea of encoding raw quantities to achieve expressive and orthogonal representations has also been studied by Gasteiger et al. for DimeNet [22], taking things further and using Bessel functions. This idea is prevalent in the literature and shows some of the complications of working with 3D coordinates.

In addition, OCP exhibits several unique characteristics that must be taken into account to extract the most performance from any given model. One such property is given by periodic boundary conditions (common for crystal systems), requiring a dedicated pre-processing step. Another characteristic is the presence of 3 types of atoms: sub-surface slab atoms, surface slab atoms, and adsorbate atoms, which must be distinguished by the model. Finally, the task chosen in the benchmarking Graphormer paper is not only relaxed energy prediction but also relaxed structure prediction, entailing the prediction of new coordinates for all atoms. This again leverages additional data in the dataset and can be considered a task with synergistic positive effects for relaxed energy prediction.

On top of this, the 3D implementation of Graphormer is not available on `huggingface` (the version used throughout the paper). We chose to perform experiments using a 10K training set that is provided as part of OCP, and the same validation set of size 25K. The entire OCP dataset consists of around 500K dense catalytic structures, and training both Graphormer and ESA/NSA on this task would entail a computational effort larger than for any other evaluated dataset. To complicate things further, each structure is dense, leading to a significant GPU memory burden such that only small batch sizes are possible even for high-end GPUs.

Moreover, with higher batch sizes we have hit a limit of PyTorch: the `nonzero()` tensor method that is used as part of the mask computation is not defined for tensors with more elements than the 32-bit integer limit. Although this operation can be chunked using smaller tensors, this induces a significant slowdown during training. Overall, this software limitation highlights the fact that current libraries are not optimised for masked attention. We present other software limitations in SI 7, as well as possible solutions, and we believe that ESA can be significantly optimised with careful software (and even hardware) design.

For the 10K train + 25K validation task, we have adapted our method to use the 3D pre-processing described above, and modified Graphormer to perform only relaxed energy prediction (i.e. without relaxed structure prediction). We used a batch size of 16 for both models, and roughly equivalent settings between NSA and Graphormer, where possible, including 4 layers, 16 attention heads, an embedding/hidden size of 256, and the same learning rate (1e-4). Both methods used mixed precision training. Here, we used the Graphormer 3D implementation from the official repository. We also note that structural information (in the form of `edge_index` tensors in PyTorch Geometric) is provided in the OCP dataset. They are derived in a similar way to PyTorch Geometric's `radius_graph()` function, which connects points based on a distance cutoff. We can use this information for ESA/NSA.

SI 13 Experimental platform

Representative versions of the software used as part of this paper include Python 3.11, PyTorch version 2.5.1 with CUDA 12.1, PyTorch Geometric 2.5.3 and 2.6.0, PyTorch Lightning 2.4.0, huggingface transformers version 4.35.2, and xFormers version 0.0.27. It is worth noting that attention masking and efficient implementations of attention are early features that are advancing rapidly. This means that their behaviour might change

unexpectedly and there might be bugs. For example, PyTorch 2.1.1 recently fixed a bug that concerned non-contiguous custom attention masks in the `scaled_dot_product_attention` function.

In terms of hardware, the GPUs used include an NVIDIA RTX 3090 with 24GB VRAM, NVIDIA V100 with 16GB or 32GB of VRAM, and NVIDIA A100 with 40GB and 80GB of VRAM. Recent, efficient implementations of attention are optimised for the newest GPU architectures, generally starting from Ampere (RTX 3090 and A100).

SI 14 Dataset statistics

We present a summary of all the used datasets, together with their size and the maximum number of nodes and edges encountered in a graph in the dataset (Supplementary Table 15). The last two are important, as they determine the shape of the mask and of the inputs for the attention blocks. Technically, we require the maximum number of nodes/edges to be determined per batch, and the tensors to be padded accordingly. This per-batch maximum is lower than the dataset maximum for most batches. However, certain operations such as layer normalisation, if performed over the last two dimensions, require a constant value. To enable this, we use the dataset maximum.

SI 15 Sourcing and licensing

Most datasets are sourced from the PyTorch Geometric library (MIT license). Datasets with different sources include: DOCKSTRING (Apache 2.0, https://github.com/dockstring/dockstring), the heterophily datasets (MIT license, https://github.com/yandex-research/heterophilous-graphs), the peptide datasets from the Long Range Graph Benchmark project (MIT license, https://github.com/vijaydwivedi75/lrgb), the GW frontier orbital energies for QM9 (CC BY 4.0 license, https://doi.org/10.6084/m9.figshare.21610077.v1), and the Open Catalyst Project (CC BY 4.0 license for the datasets, MIT license for the Python package, https://github.com/Open-Catalyst-Project/Open-Catalyst-Dataset).

All GNN implementations used in this work are sourced from the PyTorch Geometric library (MIT license). The Graphormer and TokenGT implementations are sourced from the Huggingface project (Apache 2.0 license). The Graphormer implementation used for the 3D modelling task is sourced from the official GitHub repository (MIT license, https://github.com/microsoft/Graphormer). GraphGPS also uses the MIT license. PyTorch uses the BSD-3 license.

Supplementary Table 15: Summary of used datasets, their size, and the maximum number of nodes (**N**) and edges (**E**) seen in a graph in the dataset.

	**Dataset**	**Size**	**N**	**E**
LRGB	PEPT-STRUCT	15 535	444	928
	PEPT-FUNC	15 535	444	928
NODE	PPI	24	3 480	106 754
	CORA	1	2 708	10 556
	CiteSeer	1	3 327	9 104
	ROMAN EMPIRE	1	22 662	65 854
	AMAZON RATINGS	1	24 492	186 100
	MINESWEEPER	1	10 000	78 804
	TOLOKERS	1	11 758	1 038 000
	SQUIRREL	1	2 223	93 996
	CHAMELEON	1	890	17 708
	INFECTED 15000	1	15 000	20 048
	INFECTED 30000	1	30 000	45 258
MolNet	FreeSolv	642	44	92
	LIPO	4 200	216	438
	ESOL	1 128	119	252
	BBBP	2 039	269	562
	BACE	1 513	184	376
	HIV	41 127	438	882
MOL	ZINC	249 456	38	90
	PCQM4MV2	3 452 151	51	118
NCI	NCI1	4 110	111	238
	NCI09	4 127	111	238
CV	MNIST	70 000	75	600
	CIFAR10	60 000	150	1 200
BioInf	ENZYMES	600	126	298
	PROTEINS	1 113	620	2 098
	DD	1 178	5 748	28 534
SYNTH	SYNTHETIC	300	100	392
	SYNTHETIC NEW	300	100	396
	SYNTHIE	400	100	424
SOCIAL	IMDB-BINARY	1 000	136	2 498
	IMDB-MULTI	1 500	89	2 934
	TWITCH EGOS	127 094	52	1 572
	REDDIT THR.	203 088	97	370
	QM9	133 885	29	56
	DOCKSTRING	260 060	164	342
	MalNetTiny	5 000	4 994	20 096
	Open Catalyst Project	35 000	334	11 094

SI 16 Explainability

We computed ground truth molecular orbitals using Psi4 and the same methodology as in the original QM9 paper (B3LYP functional with the 6-31G(d) basis set) [23]. The ground truth is in this case an approximation given by visualisation parameters such as the isosurface value. A lower isosurface value includes regions of lower electron density and thus a more extended/diffuse surface, while higher values would highlight only the regions with the highest density. Here, we use the default parameters in Avogadro 2.

It is worth noting that our models are trained on 2D graph representations without 3D information, and only to predict the scalar HOMO energy. As such, it would be unreasonable to expect high-quality 3D molecular orbital reconstructions. Furthermore, attention scores should be regarded as a proxy and potential explainability tool, but it is difficult if not impossible to establish a strong and reliable relationship between the actual physical coefficients and the model weights.

Nonetheless, we visualise the highest attention weights and the HOMO orbitals in Supplementary Figure 3 for several molecules and notice that the bonds and underlying atoms highlighted by attention are contained within the highlighted 3D regions. The visualisation includes molecules with varied structures and sizes. It is interesting to note that in the case of `gdb_39511` (Supplementary Figure 3C), the molecule is symmetric as far as the 2D model is concerned, and two pairs are highlighted with equal scores. The actual 3D geometry has a different structure, but the highlighted bond corresponds to the visualised orbital.

SI 17 Line graphs for graphs indistinguishable by 1-WL

Supplementary Figure 4 shows an example of two graphs, G_1 and G_2, which are indistinguishable by the 1-WL test, for example as provided by the `weisfeiler_lehman_graph_hash` function from the `networkx` library. However, the resulting line graphs (L_1 and L_2) are now distinguishable by the 1-WL test.

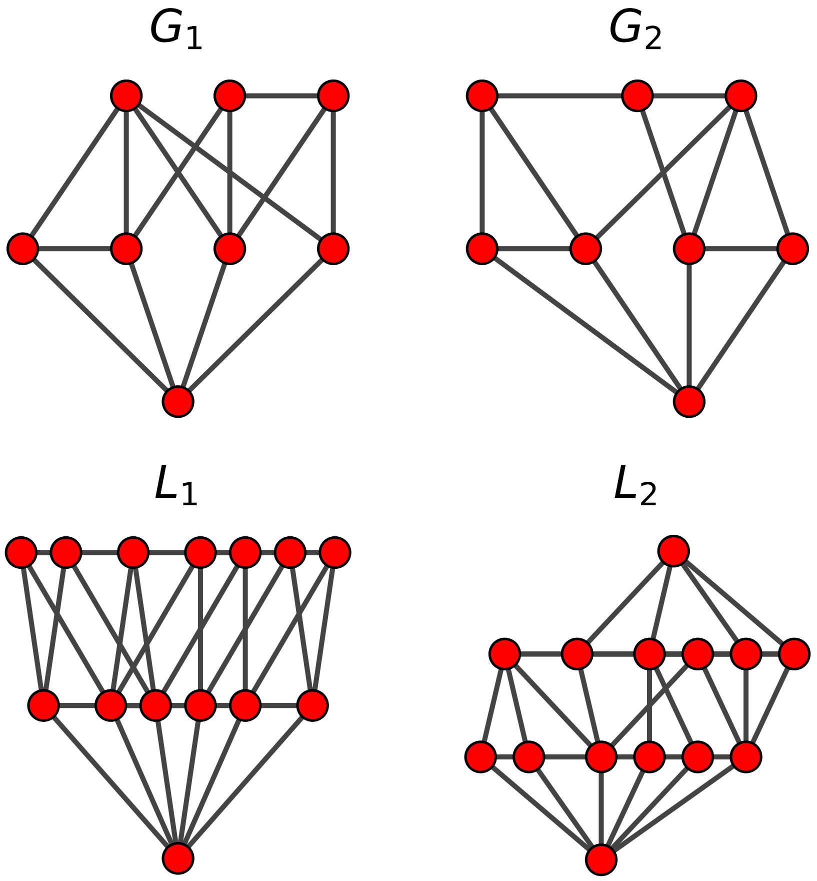

Supplementary Figure 4: Two graphs that are indistinguishable by the 1-WL test (G_1 and G_2), while their line graphs counterparts (L_1 and L_2) are distinguishable.

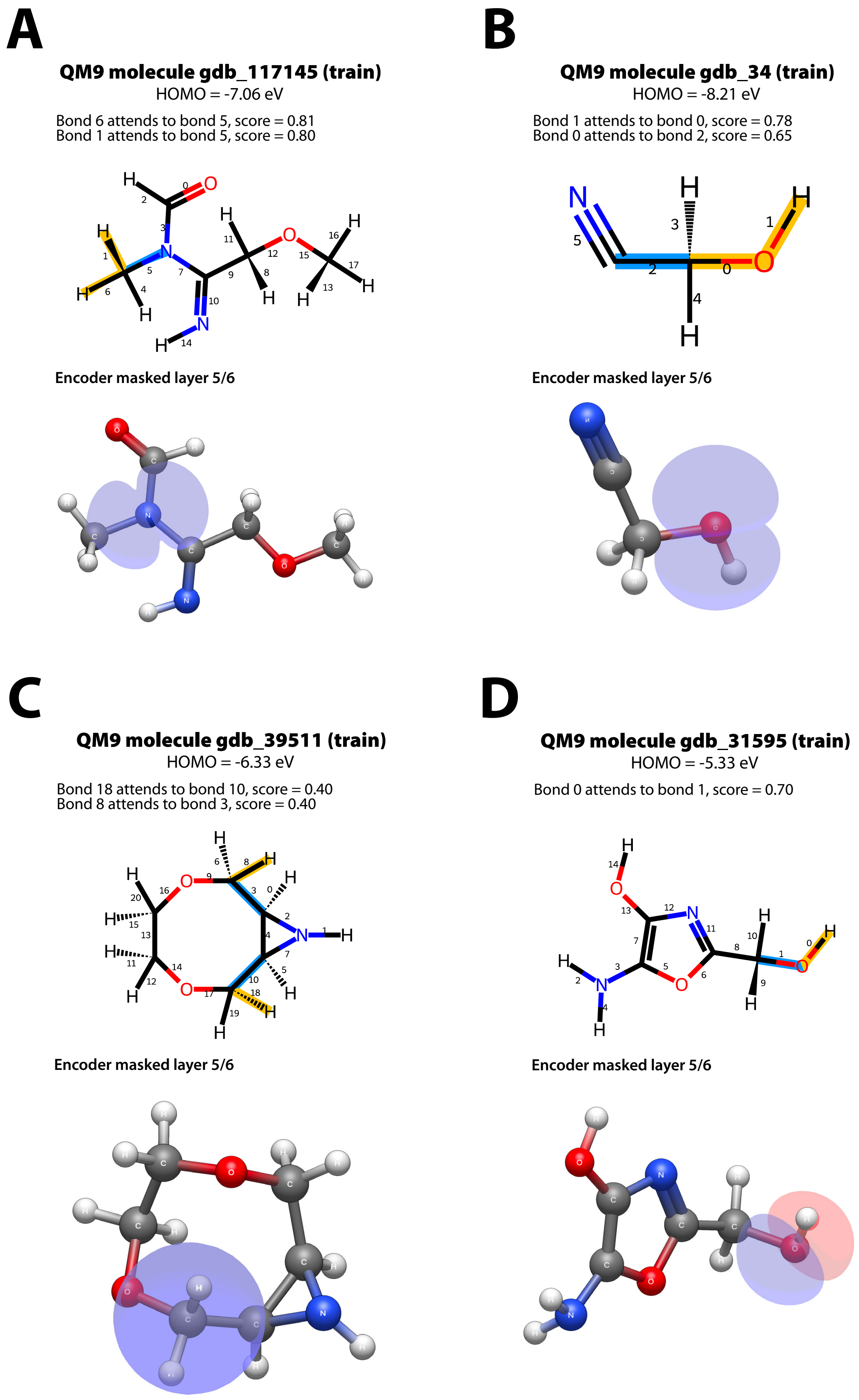

Supplementary Figure 3: A – D. Top attention weights illustrated through 2D molecular graphs and HOMO visualisations of the corresponding actual 3D geometry from the QM9 dataset. The labels for the four visualised molecules are `gdb_117145` (**A**), `gdb_34` (**B**), `gdb_39511` (**C**), `gdb_31595` (**D**).

Supplementary References

1. Kreuzer, D., Beaini, D., Hamilton, W. L., Létourneau, V. & Tossou, P. *Rethinking Graph Transformers with Spectral Attention* in *Advances in Neural Information Processing Systems* (eds Beygelzimer, A., Dauphin, Y., Liang, P. & Vaughan, J. W.) (2021).
2. Tönshoff, J., Ritzert, M., Rosenbluth, E. & Grohe, M. *Where Did the Gap Go? Reassessing the Long-Range Graph Benchmark* in *The Second Learning on Graphs Conference* (2023).
3. Shirzad, H., Velingker, A., Venkatachalam, B., Sutherland, D. J. & Sinop, A. K. *Exphormer: Sparse Transformers for Graphs* in *Proceedings of the 40th International Conference on Machine Learning* (JMLR.org, 2023).
4. Deng, C., Yue, Z. & Zhang, Z. *Polynormer: Polynomial-Expressive Graph Transformer in Linear Time* in *The Twelfth International Conference on Learning Representations* (2024).
5. Paszke, A. *et al. PyTorch: An Imperative Style, High-Performance Deep Learning Library* in *Advances in Neural Information Processing Systems 32* (Curran Associates, Inc., 2019), 8024–8035.
6. Lefaudeux, B. *et al. xFormers: A modular and hackable Transformer modelling library* https://github.com/facebookresearch/xformers. 2022.
7. Loshchilov, I. & Hutter, F. *Decoupled Weight Decay Regularization* in *International Conference on Learning Representations* (2019).
8. Dettmers, T., Lewis, M., Shleifer, S. & Zettlemoyer, L. *8-bit Optimizers via Block-wise Quantization* in *International Conference on Learning Representations* (2022).
9. Wolf, T. *et al. Transformers: State-of-the-Art Natural Language Processing* in *Proceedings of the 2020 Conference on Empirical Methods in Natural Language Processing: System Demonstrations* (eds Liu, Q. & Schlangen, D.) (Association for Computational Linguistics, Online, Oct. 2020), 38–45.
10. Luo, Y., Shi, L. & Wu, X.-M. *Classic GNNs are Strong Baselines: Reassessing GNNs for Node Classification* in *The Thirty-eight Conference on Neural Information Processing Systems Datasets and Benchmarks Track* (2024).
11. Chicco, D. & Jurman, G. The advantages of the Matthews correlation coefficient (MCC) over F1 score and accuracy in binary classification evaluation. *BMC Genomics* **21,** 6. ISSN: 1471-2164 (Jan. 2020).
12. Chicco, D., Warrens, M. J. & Jurman, G. The Matthews Correlation Coefficient (MCC) is More Informative Than Cohen's Kappa and Brier Score in Binary Classification Assessment. *IEEE Access* **9,** 78368–78381 (2021).
13. Chicco, D., Tötsch, N. & Jurman, G. The Matthews correlation coefficient (MCC) is more reliable than balanced accuracy, bookmaker informedness, and markedness in two-class confusion matrix evaluation. *BioData Mining* **14,** 13. ISSN: 1756-0381 (Feb. 2021).
14. Stoica, P. & Babu, P. Pearson–Matthews correlation coefficients for binary and multinary classification. *Signal Processing* **222,** 109511. ISSN: 0165-1684 (2024).
15. Chicco, D. & Jurman, G. The Matthews correlation coefficient (MCC) should replace the ROC AUC as the standard metric for assessing binary classification. *BioData Mining* **16,** 4. ISSN: 1756-0381 (Feb. 2023).
16. Hand, D. J. *Mismatched models, wrong results, and dreadful decisions: on choosing appropriate data mining tools* in *Proceedings of the 15th ACM SIGKDD International Conference on Knowledge Discovery and Data Mining* (Association for Computing Machinery, Paris, France, 2009), 1–2. ISBN: 9781605584959.
17. Kwegyir-Aggrey, K., Gerchick, M., Mohan, M., Horowitz, A. & Venkatasubramanian, S. *The Misuse of AUC: What High Impact Risk Assessment Gets Wrong* in *Proceedings of the 2023 ACM Conference on Fairness, Accountability, and Transparency* (Association for Computing Machinery, Chicago, IL, USA, 2023), 1570–1583. ISBN: 9798400701924.
18. Chicco, D., Warrens, M. J. & Jurman, G. The coefficient of determination R-squared is more informative than SMAPE, MAE, MAPE, MSE and RMSE in regression analysis evaluation. en. *PeerJ Comput Sci* **7,** e623 (July 2021).
19. Brody, S., Alon, U. & Yahav, E. *How Attentive are Graph Attention Networks?* in *International Conference on Learning Representations* (2022).
20. Wang, Q., Chen, D. Z., Wijesinghe, A., Li, S. & Farhan, M. *$\mathscr{N}$-WL: A New Hierarchy of Expressivity for Graph Neural Networks* in *The Eleventh International Conference on Learning Representations* (2023).
21. Shi, Y. *et al. Benchmarking Graphormer on Large-Scale Molecular Modeling Datasets* 2023. arXiv: 2203.04810 [cs.LG].
22. Gasteiger, J., Groß, J. & Günnemann, S. *Directional Message Passing for Molecular Graphs* in *International Conference on Learning Representations (ICLR)* (2020).
23. Ramakrishnan, R., Dral, P. O., Rupp, M. & von Lilienfeld, O. A. Quantum chemistry structures and properties of 134 kilo molecules. *Scientific Data* **1,** 140022. ISSN: 2052-4463 (Aug. 2014).
